# CA-30, an oligosaccharide fraction derived from Liuwei Dihuang decoction, ameliorates cognitive deterioration via the intestinal microbiome in the senescence-accelerated mouse prone 8 strain

**DOI:** 10.18632/aging.101990

**Published:** 2019-06-03

**Authors:** Jianhui Wang, Xi Lei, Zongjie Xie, Xiaorui Zhang, Xiaorui Cheng, Wenxia Zhou, Yongxiang Zhang

**Affiliations:** 1 Beijing Institute of Pharmacology and Toxicology, Beijing 100850, China; 2 State Key Laboratory of Toxicology and Medical Countermeasures, Beijing 100850, China

**Keywords:** Alzheimer’s disease, CA-30, oligosaccharide, senescence-accelerated mouse prone 8 strain, intestinal microbiome

## Abstract

Mounting evidence points to alterations in the gut microbiota-neuroendocrine immunomodulation (NIM) network that might drive Alzheimer’s Disease (AD) pathology. In previous studies, we found that Liuwei Dihuang decoction (LW) had beneficial effects on the cognitive impairments and gastrointestinal microbiota dysbiosis in an AD mouse model. In particular, CA-30 is an oligosaccharide fraction derived from LW. We sought to determine the effects of CA-30 on the composition and function of the intestinal microbiome in the senescence-accelerated mouse prone 8 (SAMP8) mouse strain, an AD mouse model. Treatment with CA-30 delayed aging processes, ameliorated cognition in SAMP8 mice. Moreover, CA-30 ameliorated abnormal NIM network in SAMP8 mice. In addition, we found that CA-30 mainly altered the abundance of four genera and 10 newborn genera. Advantageous changes in carbohydrate-active enzymes of SAMP8 mice following CA-30 treatment, especially GH85, were also noted. We further found that seven genera were significantly correlated with the NIM network and cognitive performance. CA-30 influenced the relative abundance of these intestinal microbiomes in SAMP8 mice and restored them to SAMR1 mouse levels. CA-30 ameliorated the intestinal microbiome, rebalanced the NIM network, improved the AD-like cognitive impairments in SAMP8 mice, and can thus be a potential therapeutic agent for AD.

## INTRODUCTION

Alzheimer’s disease (AD) is a common, age-related neurodegenerative disorder with complex etiologies including genetics and other external environmental components [1]. In patients with AD, aggregates of amyloid-β (Aβ) peptides and *tau* protein phosphorylation in the central nervous system impair cognitive function [2]. How intrinsic and external factors regulate these processes remains unclear, however.

Accumulating evidence supports the notion that alterations in the intestinal bacteria-neuroendocrine immunomodulation (NIM) network may contribute to the pathogenesis of AD [3–5]. For instance, amyloidosis may be regulated by the production of amyloid peptides by host microbiota [6], with the Aβ peptide is relayed via myenteric neurons and spreads to the central nervous system via the gut-brain axis [7]. Further supporting the role of gut microbes in AD, Aβ deposition in PrP-hAβPPswe/PS1^ΔE9^ transgenic mice was reduced by broad-spectrum antibiotic treatment [8]. These results are consistent with reduced cerebral and serum Aβ levels in a mouse model of AD reared under germ-free conditions [9]. Moreover, manipulating the intestinal microbiota with antibiotics [10] or via probiotic therapy [11] alters cognition, such as novel object recognition, spatial learning and memory. Despite these results, the role of intestinal bacteria-immune directed pathways in the pathogenesis of AD requires further interrogation with integrated and holistic approaches [12].

Many oligosaccharides are not digested by humans, as the human body lacks the enzymes required to hydrolyze the β-links formed among some monosaccharide units [13]. Most oligosaccharides are quantitatively hydrolyzed to small oligomers and monomers by glycosidases or glycosyl transferases in the upper part of the gastrointestinal tract [14]. The resulting monosaccharides are transported *via* the portal blood to the liver and, subsequently, to the systemic circulation [15]. When these oligosaccharides are consumed, the undigested fraction serves as food for beneficial bacteria, such as species of *Bifidobacteria* and *Lactobacillus* [16, 17].

CA-30, an oligosaccharide fraction derived from Liuwei Dihuang decoction (LW) as holistic medicine, mainly consists of stachyose and mannotriose [18]. Studies have reported that LW and its active fractions have beneficial effects on AD-like cognitive impairments. In the senescence-accelerated mouse prone 8 strain (SAMP8), one of the most widely accepted murine models for the study of late-onset/age-related AD etiopathogenesis [19, 20], the chronic administration of LW or its active fractions was found to significantly ameliorate declines in learning and memory performance [18, 21–23]. Other studies further demonstrated that LW and its active fractions have advantageous effects on neurodegenerative pathology [24–28]. Furthermore, LW and its active fractions have restorative and modulatory effects on the neuroendocrine-immune system and intestinal microbiome in AD animal models [29, 30]. To investigate the mechanisms underlying the anti-AD effects of oligosaccharide LW fractions, we assessed the effects of CA-30 on cognitive impairments via modulation of the intestinal microbiome. To study this, we examined the effects of CA-30 on cognitive performance, NIM network mediation, and the composition and function of gut microbiota in SAMP8 mice.

## RESULTS

### CA-30 slowed aging in SAMP8 mice

**Figure 1:**
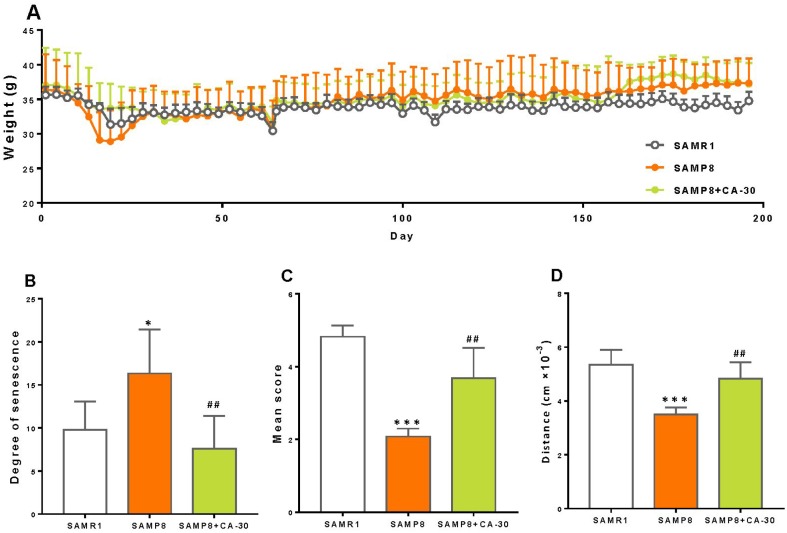
**CA-30 slowed aging in SAMP8 mice.** The weight (**A**), degree of senescence (**B**), nest building score (**C**), and spontaneous locomotor activity (**D**) of SAMR1, SAMP8, and SAMP8 CA-30-treated mice. *P<0.05, ***P<0.001, versus SAMR1 mice, ^##^*P*<0.01, versus SAMP8 mice by unpaired Student's *t*-tests. All values are means ± S.D. n=9-11.

SAMP8 mice are a model of accelerated senescence. Our results demonstrate that the body weight of SAMP8 mice did not differ significantly from SAMR1 or CA-30 treated animals (Figure 1A). SAMP8 mice exhibited a greater degree of senescence than did SAMR1 mice (Figure 1B) but lower mean nest building and total spontaneous locomotor activity than SAMR1 mice (Figure 1C and 1D). After being treated with CA-30, senescence in SAMP8 mice significantly decreased and their nest building and locomotor activity increased. This indicates that treatment with CA-30 delays aging processes in SAMP8 mice.

### CA-30 improved cognitive impairments in SAMP8 mice

**Figure 2:**
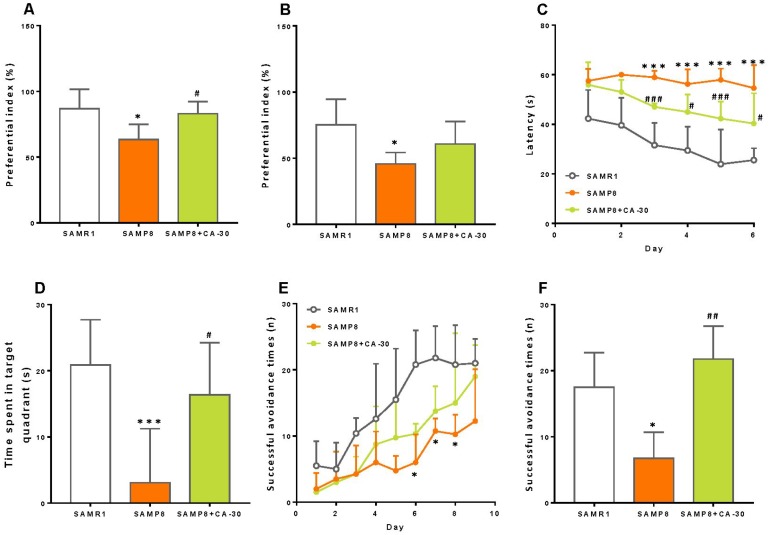
**CA-30 improved cognitive impairments in SAMP8 mice.** The preferential index (time on novel object C/(time on novel object C+time on sample object A)×100%) after 1 hr (**A**) and 24 hr (**B**) of training in the testing phase of the novel object recognition test. Latency to animals’ first crossing the platform location during hidden-platform training (**C**). Time spent swimming in the target quadrant during the probe trial (**D**). Successful avoidance times in the training (**E**) and testing sessions (**F**) *P<0.05, ***P<0.001, versus the SAMR1 mice, ^#^*P*<0.05, ^##^*P*<0.01, ^###^*P*<0.001, versus the SAMP8 mice by unpaired Student's *t*-tests and two-way repeated-measures analyses of variance with Tukey multiple comparisons tests. All values are means ± S.D. n=9-11.

The results of novel object recognition testing revealed a significantly decreased preferential index among SAMP8 mice in both the short- and long-term (Figure 2A and 2B). Treatment with CA-30 significantly improved these animals’ short-term memory, however (Figure 2A). This indicates that short-term object recognition memory deterioration in SAMP8 mice may be reversed by CA-30 treatment.

To examine spatial learning and memory in these mice, we employed the Morris water maze test. In this task, the SAMP8 group exhibited a longer escape latency than did the SAMR1 group beginning on the third day of testing. Furthermore, administration of CA-30 decreased this latency in the SAMP8 group (Figure 2C). In the probe trial, swimming time within the target quadrant was significantly decreased in the SAMP8 group compared to the SAMR1 group, while CA-30 normalized this time in the SAMP8 group (Figure 2D). The number of platform crosses and latency to reaching the platform in the probe trial was not significantly different between the SAMP8 and SAMR1 groups (data not shown). This indicates that CA-30 has a protective effect on spatial learning and memory impairments in SAMP8 mice.

Finally, the shuttle-box test was used to evaluate active avoidance in mice in the present study. Successful avoidance times in SAMP8 group were significantly decreased beyond SAMR1 group levels beginning on the sixth day of training (Figure 2E). Successful testing phase avoidance times in the SAMP8 group also decreased as compared to the SAMR1 group. These avoidance times increased, however, after the CA-30 administration (Figure 2F). This indicates that CA-30 ameliorates active avoidance deficits in SAMP8 mice.

### CA-30 balanced the neuroendocrine immunomodulation system in SAMP8 mice

**Figure 3:**
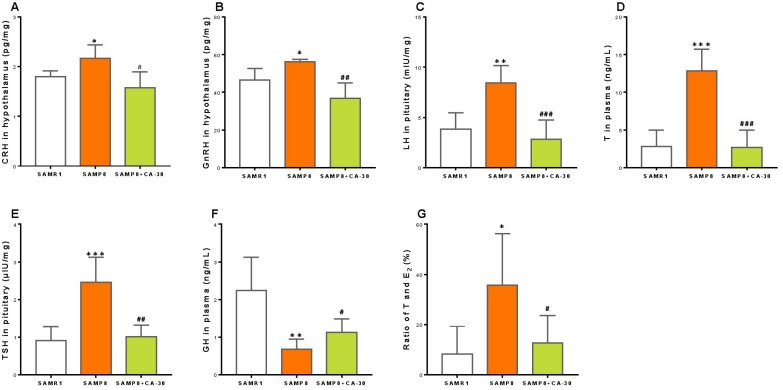
**CA-30 restored the secretion of endocrine hormones in SMAP8 mice.** Concentrations of corticotropin-releasing hormone (CRH) (**A**), gonadotropin-releasing hormone (GnRH) (**B**), luteinizing hormone (LH) (**C**), testosterone (T) (**D**), thyrotropic hormone-releasing hormone (TSH) (**E**), growth hormone (GH) (**F**) in mice, and the concentration ratio of T and estradiol (E_2_) (**G**). *P<0.05, **P<0.01, ***P<0.001, versus the SAMR1 mice, ^#^*P*<0.05, ^##^*P*<0.01, ^###^*P*<0.001, versus the SAMP8 mice by unpaired Student's *t*-tests. All values are means ± S.D. n=9-11.

To investigate neuroendocrine system functioning in SAMP8 mice and any potential influence of the CA-30, the concentrations of CRH, GnRH, and TRH in the hypothalamus; ACTH, FSH, LH, TSH, and GH in the pituitary; and corticosterone, T, E_2_, T3, and T4 in the plasma were measured by radioimmunoassay. We found that the concentration of CRH (a central player in the HPA axis) was significantly increased in the SAMP8 group as compared to the SAMR1 group and significantly decreased with CA-30 administration (Figure 3A). Within the HPG axis, the concentrations of GnRH, LH, and T were an increased in the SAMP8 group and declined significantly with CA-30 administration (Figure 3B–3D). Within the HPT axis, the level of TSH increased in the SAMP8 group, while CA-30 significantly decreased TSH levels (Figure 3E). In addition, the concentration of GH in the plasma was significantly lower in the SAMP8 group than in the SAMR1 group and was increased by CA-30 administration (Figure 3F). The concentrations of ACTH and corticosterone in the HPA axis; FSH and E_2_ in the HPG axis; and TRH, T3, and T4 in the HPT axis were not significantly different between the SAMP8 and SAMR1 groups (data not shown). Nevertheless, decreases in the ratio of T to E_2_ in the plasma were observed in the SAMP8 group compared to the SAMR1 group. CA-30 treatment significantly restored this ratio to control levels (Figure 3G). These data indicate that CA-30 ameliorated multiple neuroendocrine system phenotypes in SAMP8 mice.

**Figure 4:**
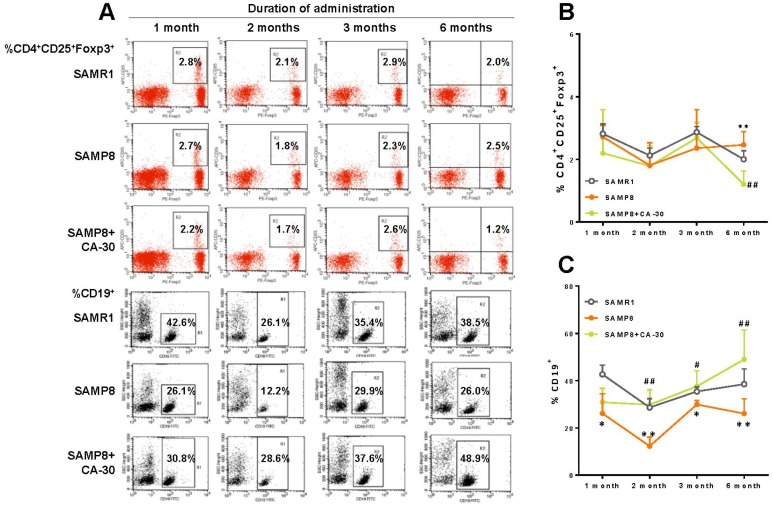
**CA-30 corrected aberrant lymphocyte subsets in SAMP8 mice.** Representative flow cytometric plots for CD4^+^CD25^+^Foxp3^+^ T cells and CD19^+^ B cells (**A**). Flow cytometric quantification of CD4^+^CD25^+^Foxp3^+^ T cells (**B**) and CD19^+^ B (**C**) cells in mouse blood. Approximately 5 × 10^5^ whole blood cells for each mouse were harvested, washed, and incubated with antibodies, then quantified via flow cytometry. *P<0.05, **P<0.01, versus the SAMR1 mice, ^#^*P*<0.05, ^##^*P*<0.01, versus the SAMP8 mice by unpaired Student's *t*-tests and two-way repeated-measures analyses of variance with Tukey multiple comparisons tests. All values are means ± S.D. n=9-11.

To investigate the effects of CA-30 on lymphocyte subsets in SAMP8 mice, the dynamic expression of CD3^+^ T cells, CD3^+^CD4^+^ T cells, CD3^+^ CD8^+^ T cells, CD8^+^ CD28^+^ T cells, CD4^+^ CD25^+^Foxp3^+^T cells, and CD19^+^ B cells one, two, three, and six months after CA-30 administration were assessed by flow cytometry. The expression of CD4^+^CD25^+^Foxp3^+^T (Treg) cells in the SAMP8 group was significantly increased over SAMR1 group levels in year-old mice (six months after CA-30 administration) (Figure 4B). The expression of CD19^+^ B cells in the SAMP8 group was significantly decreased in the SAMR1 group in seven-month-old mice (one month after administration CA-30) (Figure 4C). Expression of the other lymphocyte subsets in SAMP8 mice (data not shown) was not significantly changed by CA-30. In the SAMP8 group, the expression of Treg cells decreased after six months of CA-30 treatment (Figure 4B), while the expression of CD19^+^ B cells increased after two months of CA-30 treatment (Figure 4C). These results indicate that CA-30 treatment partially restored normal lymphocyte expression in SAMP8 mice.

**Figure 5:**
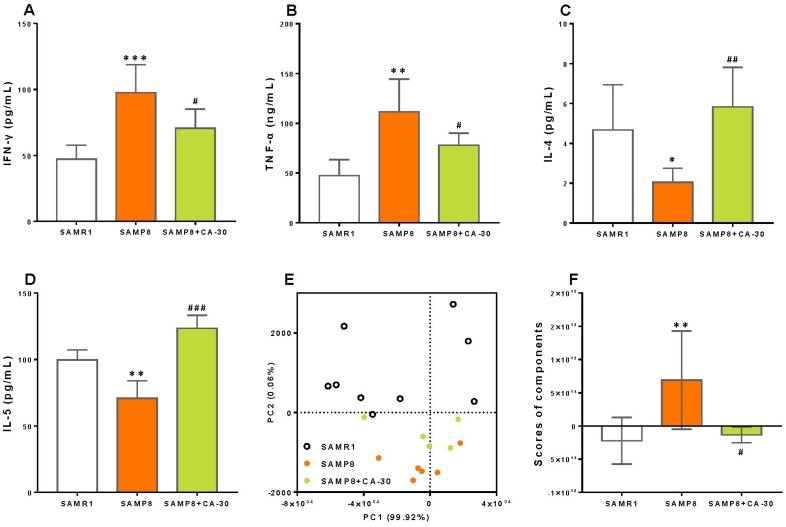
**CA-30 regulated abnormal cytokine secretion in SAMP8 mice.** Concentrations (pg/mL) of interferon-γ (IFN-γ) (**A**), tumor necrosis factor α (TNF-α) (**B**), interleukin-4 (IL-4) (**C**) and interleukin-5 (IL-5) (**D**) in the blood plasma of mice were detected using Luminex® X-MAP® technology. Principal component analysis (PCA) of cytokine secretion of mice (**E**), with each point representing one mouse. PCA conducted with SAS 9.2 statistics package, with significance set at *P* < 0.05. Average PCA scores (**F**). *P<0.05, **P<0.01, ***P<0.001, versus the SAMR1 mice, ^#^*P*<0.05, ^##^*P*<0.01, ^###^*P*<0.001, versus SAMP8 mice by unpaired Student's *t*-tests. All values are means ± S.D. n=9-11.

To assessed the effects of CA-30 on cytokine secretion, a multiplex bead analysis approach was employed to detect the concentration of anti-inflammatory (IL-4, IL-5, IL-10, and G-CSF) and pro-inflammatory (IL-1β, IL-2, IL-6, IL-17, IL-23, IFN-γ, TNF-α, TNF-β, GM-CSF, Eotaxin, RANTES, MCP-1, and MIP-1β) cytokines in the blood plasma of mice. Increased levels of IFN-γ (Figure 5A) and TNF-α (Figure 5B) and decreased levels of IL-4 (Figure 5C) and IL-5 (Figure 5D) in the blood plasma were observed in the SAMP8 group as compared to the SAMR1 group. Levels of 13 other cytokines (data not shown) remained unchanged.

CA-30 treatment significantly decreased the production of IFN-γ and TNF-α and increased levels of IL-4 and IL-5 in the present study. To determine the effects of CA-30 on abnormal cytokine secretion in SAMP8 mice, principal component analysis (PCA) was performed here. PCA utilized data about the secretion of 17 cytokines in the SAMR1, SAMP8, and CA-30-treated groups and revealed that principal components 1 (PC1) and 2 (PC2) grouped mice from the three different groups into three distinct clusters (Figure 5E). Mice in the SAMR1 group were localized to the top of the resultant PCA graph, most of the mice in the SAMP8 group were localized to the bottom, and mice in the CA-30 group were localized between the SAMR1 and SAMP8 groups. To further analyze the effects of CA-30 on cytokine secretion, average PCA scores were graphically depicted (Figure 5F). This depiction revealed that the average cytokine score of the SAMP8 group was significantly higher than that of the SAMR1 group, and administration of CA-30 decreased this score. These results indicate that cytokine secretion in SAMP8 mice was abnormal and that administration of CA-30 restored this aberrant immune phenotype inSAMP8 mice.

### CA-30 restored gut microbiota imbalances in SAMP8 mice

The effects of CA-30 on gut microbiota were determined via metagenomic sequencing of DNA extracted from fecal specimens. At the phylum level, *Bacteroidetes* (48.4% on average), *Firmicutes* (43.2% on average), and *Proteobacteria* (4.5% on average) were the dominant bacterial taxa. There were no significant differences in the abundance of *Firmicutes* and *Bacteroidetes* (F/B) between the groups (Supplementary Figure 1). A Venn diagram indicated that 1175 (96.9%) genera were common to all mice, while four (0.03%) genera appeared only in the SAMR1 group, four (0.03%) in the SAMP8 group, and 10 (0.08%) in the CA-30 group. The greatest number of unique genera were identified in the CA-30 group (Supplementary Figure 2).

**Figure 6:**
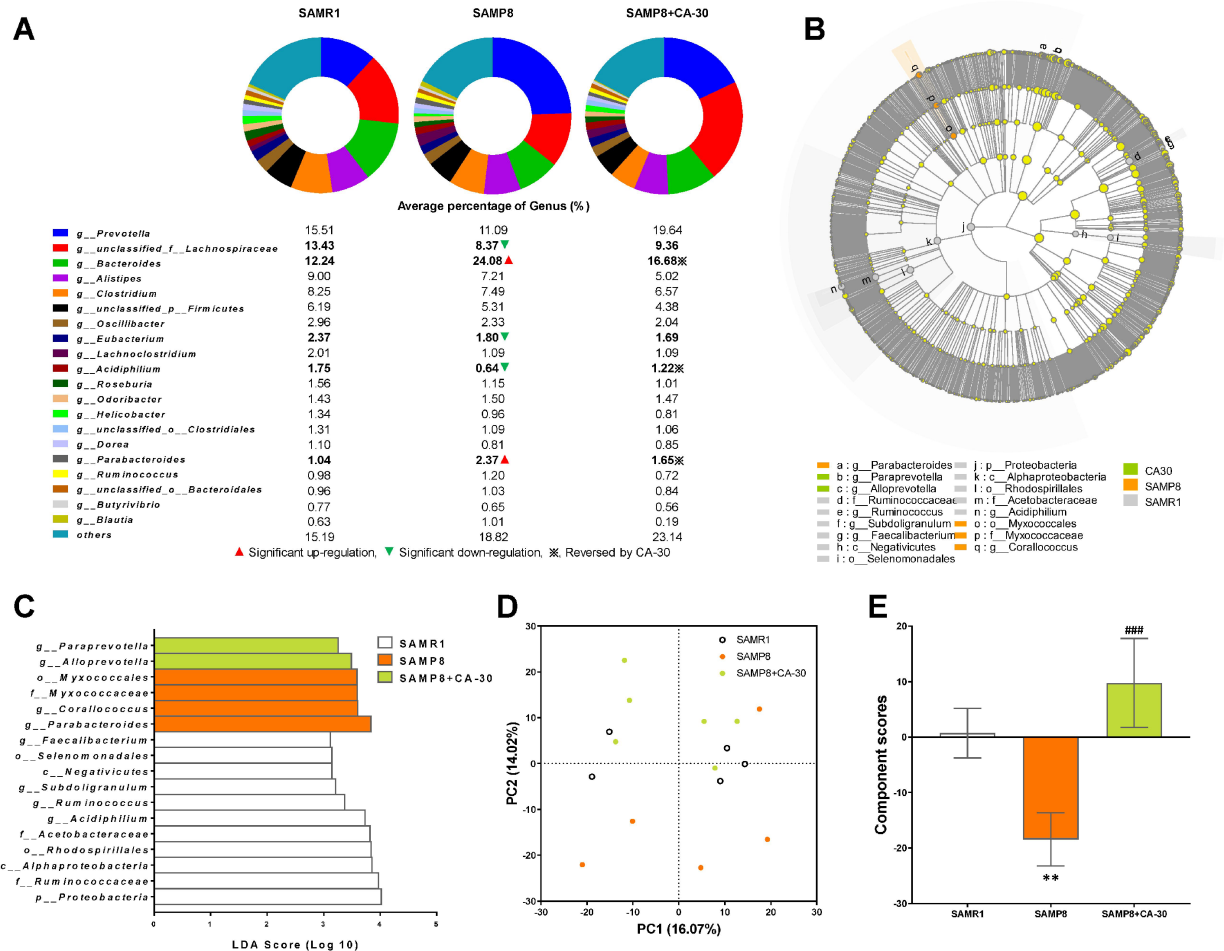
**Effects of CA-30 on the abundance and diversity of gut microbiota.** The relative abundance of the top 20 dominant gut bacterial genus in each group (**A**). The total abundance of other genera was less than 1%. ▲,▼, and ※ indicate a significant increase or decrease, respectively, in SAMP8 mice relative to SAMR1 mice, and SAMP8 mice administrated with CA-30 relative to SAMP8 mice. The enriched taxa in the gut microbiota of mice are represented in cladograms (**B**). The central point represents the root of the tree (bacteria), and each ring represents the next lower taxonomic level (phylum to genus). The diameter of each circle represents the relative abundance of the taxon. The most differentially abundant taxa in each group identified by linear discriminant analysis (LDA) scores generated from the Linear discriminant analysis effect size (LEfSe) analysis (**C**). Weighted principal component analyses (PCA) at the Genus level in each group (**D**) and average PCA scores (**E**). *P<0.05, **P<0.01, versus the SAMR1 mice, ^###^*P*<0.001, versus the SAMP8 mice by unpaired Student's *t*-tests. All values are means ± S.D. n=5-6.

At the genus level, *Prevotella, Lachnospiraceae, Bacteroides, Clostridium, Alistipes, Firmicutes, Oscillibacter, Eubacterium, Parabacteroides, Odoribacter, Lachnoclostridium, Roseburia, Acidiphilium, Clostridiales, Helicobacter, Ruminococcus, Bacteroidales, Dorea, Butyrivibrio*, and *Blautiawere* were highly abundant (Figure 6A). Moreover, the percent of community abundance of *Lachnospiraceae*, *Eubacterium*, and *Acidiphilium* in the SAMP8 group was lower than in the SAMR1 group. CA-30 decreased the percents of *Bacteroides*, *Acidiphilium*, and *Parabacteroides* and increased the percents of *Bacteroides* and *Parabacteroides* (Figure 6A). There were only four genera unique to the SAMR1 and CA-30 treated mice, including *Thermomonas, Paramesorhizobium, Alicycliphilus,* and *Candidatus_Midichloria*. Four genera in SAMP8 mice were eliminated by CA-30 administration (*Malonomonas, Nevskia, Candidatus_Megaira, and unclassified_f_Chromobacteriaceae*). Ten newborn genera were present only in CA-30 treated mice, including *Xylophilus, Serpens, Hathewaya, Tanticharoenia, Wigglesworthia, Kinetoplasti bacterium, Ventosimonas, Marinosulfonomonas, unclassified_c_Epsilonproteobacteria, and unclassified_o_Desulfovibrionales*.

Linear discriminant analysis effect size (LEfSe) testing was used to further assess alterations in microbiota composition with CA-30 treatment. The structure and predominant microbiota in each group were also represented as a cladogram (Figure 6B). The greatest differences in taxa from the phylum to the genus level was identified by linear discriminant analysis (LDA) scoring (Figure 6C). Most specific taxa in the SAMP8 group were mainly from the *Proteobacteria* phyla (75% on average). The SAMR1 group contained two dominant phyla: *Firmicutes* (45.5% on average) and *Proteobacteria* phyla (36.4% on average). The CA-30 group contained only taxa from the *Bacteroidetes* phyla. Weighted UniFrac-based PCA revealed distinct clusters of microbiota in the CA-30-treated groups (Figure 6D). Average PCA scores revealed that the average gut microbiota score of the SAMP8 group was significantly lower than that of the SAMR1 group, while administration of CA-30 increased this score (Figure 6E). These results suggested that CA-30 facilitates advantageous changes in the structure of the gut microbial community.

**Figure 7:**
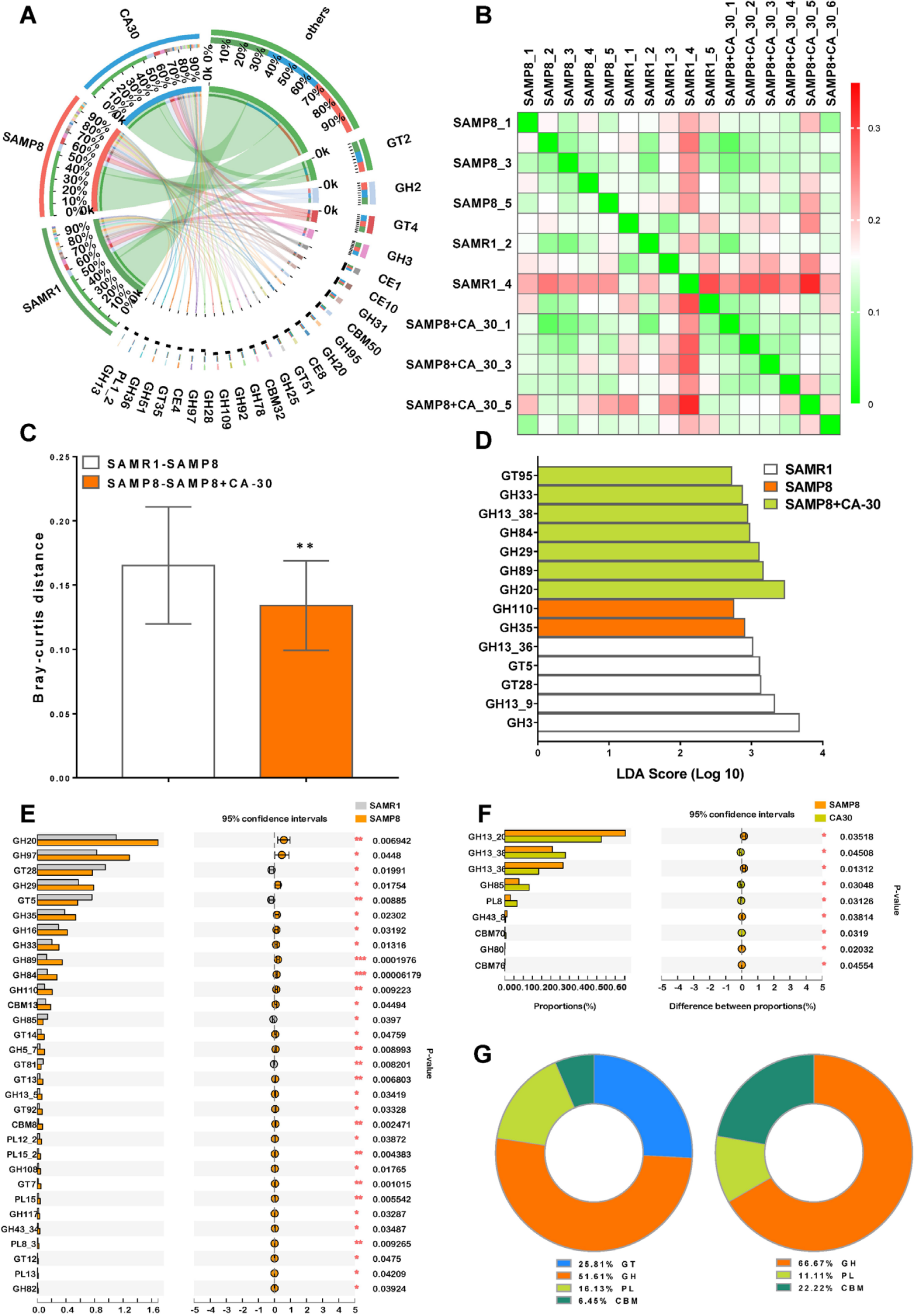
**CA-30 modulated the genetic capacity for carbohydrate utilization of gut microbiota.** Circos plot of the glycosyltransferases (GT), glycoside hydrolase (GH), polysaccharide lyase (PL), carbohydrate-binding module (CBM), and carbohydrate esterase (CE) families of carbohydrate degrading enzymes found in the metagenome using the carbohydrate-active enzyme (CAZy) database and percent of community abundance of the CAZy family level (**A**). Bray-Curtis distance heat map at the CAZy family level with the highest frequency and relative abundance, as calculated via the unweighted pair group method with arithmetic means (**B**) and Bray-Curtis distances among the mice (**C**). **P<0.01, versus the SAMR1-SAMP8 mice by unpaired Student's *t*-tests. The most differentially abundant CAZy family in each group was identified by linear discriminant analysis scores generated via linear discriminant analysis effect size analyses (**D**). Comparison of the relative abundances of the dominant CAZy Family in all groups (**E** and **F**). *P<0.05, **P<0.01, ***P<0.01, versus the SAMR1 or SAMP8 mice by unpaired Student's *t*-tests. The ratio of significantly changed CAZy classes between the SAMR1 and SAMP8 groups and the SAMP8 and CA-30 groups (**G**). All values are means ± S.D. n=5-6.

The carbohydrate-active enzyme (CAZy) database is a knowledge-based resource specialized in the enzymes that build and breakdown complex carbohydrates and glycoconjugates [31]. CAZy-encoding genes were identified and grouped into 418 CAZy gene families. To the best of our knowledge, this is the first report to analyze CAZy in the gut metagenome of SAM mice. A Venn diagram indicated that 387 (92.6%) CAZy families were common among all of the mice, with the highest number of unique CAZy families (5, 1.2%) identified in the CA-30 group (Supplementary Figure 3). Twenty-five carbohydrate-active enzymes were revealed in a condensed Circos-based visualization. The main source of these enzymes were five dominant CAZy families: the glycoside hydrolase (GH) (14, 56%), glycosyltransferases (GT) (4, 16%), carbohydrate esterase (CE) (4, 16%), carbohydrate-binding module (CBM) (2, 8%), and polysaccharide lyase (PL) (1, 4%) families (Figure 7A).

Hierarchical clustering analyses of enriched CAZy families were represented as a heat map (Figure 7B). We observed significant Bray-Curtis distance differences between the SAMR1 and SAMP8 groups and the SAMP8 and CA-30 groups on the basis of CAZy family profiles (Figure 7C). The greatest CAZy family difference in each group was identified in the GH family via LDA scores (Figure 7D). When compared to the SAMR1 group, there were 27 significantly increased and four decreased CAZy families in the SAMP8 group (Figure 7E). When SAMP8 mice were treated with CA-30, nine CAZy families were significantly altered (four increased and five decreased) (Figure 7F). Among these, the percent of community abundance of GH85 in the SAMP8 group was lower than in the SAMR1 group, while the abundance of GH85 was reversed considerably by CA-30 treatment. Similar to our LEfSe analysis findings (Figure 7D), the most significantly changed CAZy families between groups were concentrated in the GH family (Figure 7G). Collectively, these data suggest that CA-30 treatment facilitated advantageous SAMR1-like changes in gut microbial function in SAMP8 mice and that this amelioration might be principally relevant to the modulation of GH family-related changes.

### CA-30 cognition, NIM network, and gut microbiome associations in SAMP8 mice

**Figure 8:**
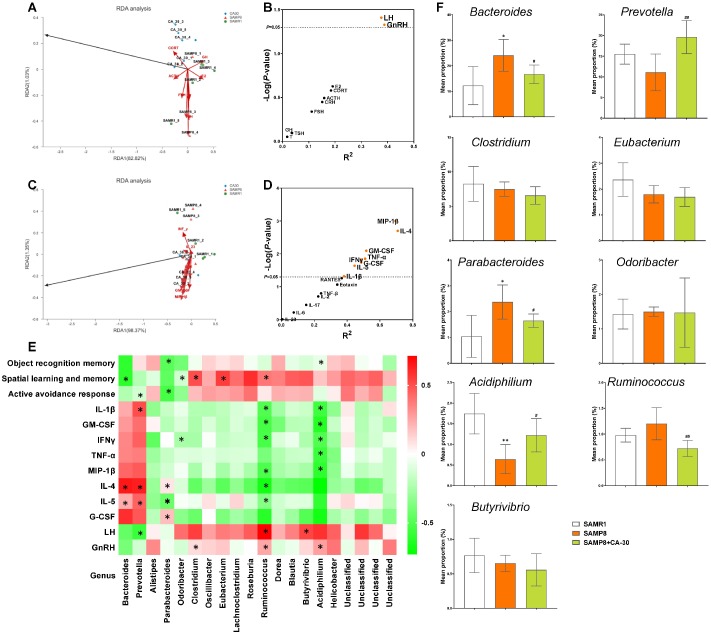
**Relationships among cognition, NIM network, and gut microbiome in SAMP8 mice.** Redundancy analysis (RDA) of the relationships among the main bacterial Genus, endocrine system hormones (**A**), and immune system cytokines (**C**). Correlations between hormones (**B**), cytokines (**D**), and main bacterial genus per redundancy analysis. The X and Y axes were derived from R^2^ and -Log (*P*), respectively. Bacterial taxa in genus correlated with cognitive performance and levels of hormones and cytokines (*P<0.05 in redundancy analysis) in SAMP8 mice (**E**). Comparison of the relative abundances of the dominant genus (correlated with NIM network or cognitive performance) in each group (**F**). *P<0.05, versus the SAMR1 mice, ^#^*P*<0.05, ^##^*P*<0.01, versus the SAMP8 mice by Kruskal-Wallis H tests and post-hoc Scheffe's tests. All values are means ± S.D. n=5-6.

We further assessed correlations between altered bacterial genus, hormones/cytokines in the NIM network, and cognitive abilities in all mice via redundancy and Spearman correlation analyses. Redundancy analyses of hormones/cytokines and bacterial genus revealed that the explanatory variables accounted for 83.85% (Figure 8A) and 99.72% (Figure 8C) of the total variation in each, respectively. The concentrations of the pro-inflammatory cytokines IL-1β, IFN-γ, TNF-α, GM-CSF, and MIP-1β and the anti-inflammatory cytokines IL-4, IL-5, and G-CSF were the primary positive environmental contributors to microbiota composition in mice from all groups (Figure 8B), but were negatively correlated with HPG axis levels of GnRH and LH (Figure 8D).

Spearman correlation analyses revealed that, at the genus level, there was eight bacterial genera correlated with levels of hormones/cytokines in the NIM network, including *Bacteroides*, *Prevotella*, *Clostridium*, *Parabacteroides*, *Odoribacter*, *Acidiphilium*, *Ruminococcus*, and *Butyrivibrio* (Figure 8E). Eight genera were correlated with cognitive abilities, including two negatively correlated (*Parabacteroides* and *Acidiphilium*) with object recognition memory, two negatively correlated (*Bacteroides* and *Odoribacter*) and three positively correlated (*Clostridium*, *Eubacterium* and *Ruminococcus*) with spatial learning and memory, and two negatively correlated (*Prevotella* and *Parabacteroides*) with the active avoidance response. Of these, *Parabacteroides* was significantly negatively correlated with cognitive performance in the novel object recognition and shuttle-box tests (Figure 8E).

Furthermore, comparisons of the dominant genus (correlated with NIM network or cognitive performance) revealed significant increases in the relative abundances of *Bacteroides* and *Parabacteroides* and significant decreases in the relative abundances of *Acidiphilium* in the SAMP8 group as compared to the SAMR1 group. CA-30 treatment significantly decreased the relative abundances of *Bacteroides* and *Parabacteroides* and increased the relative abundance of *Acidiphilium*. In addition, CA-30 treatment significantly modulated the relative abundances of *Prevotella* and *Ruminococcus*, which were not significantly different between the SAMP8 and SAMR1 groups (Figure 8F). These data suggest that CA-30 treatment might ameliorate cognitive impairments by modulating intestinal microbial composition and function of SAMP8 mice.

## DISCUSSION

In this present study, we found that CA-30 was associated with a series of gut-brain axis events in an AD mouse model, including markedly slowed aging, improved cognition, restoration of hormone and cytokine secretion in the NIM network, diversity and steady-state composition of the microbial community, and CAZymes in the intestinal microbiome. We hypothesized that CA-30 might ameliorate some AD-like cognitive impairments by modulating the intestinal microbiome.

Metabolites from the fermentation of complex carbohydrates can benefit health [32], and changes in these metabolites may further lead to changes in the levels of carbohydrate-active enzymes. Glycoside hydrolases of CAZymes play a key role in a variety of biological processes closely related to neurodegenerative diseases, such as AD [33]. The microtubule-associated protein tau (*tau*) and the amyloid precursor protein (APP) are two proteins that give rise to the chief pathological hallmarks of AD. Post-translational modifications of *tau* and APP are of considerable interest given that such modifications alter the neurotoxicity of these proteins. Such post-translational modifications are also critically implicated in AD pathology [34, 35]. Among modified proteins, both APP and *tau* are enzymatically modified with *N*-acetyl-D-glucosamine (GlcNAc) residues *O*-linked to the hydroxyl groups of serine and threonine residues (*O*-GlcNAc) [36]. *O*-GlcNAc is cleaved from modified proteins by a glycoside hydrolase called *O*-GlcNAcase (OGA), which belongs to GH84 (EC 3.2.1.169).

A large body of evidence further suggests a causal relationship between impaired brain glucose metabolism and neuronal damage in AD [37–39]. In fact, cerebral glucose hypometabolism precedes the appearance of AD clinical symptoms [40] and decreased concentrations of GT type 1 and 3 have been found in the cerebral cortex of patients with AD [41]. Moreover, this dysregulation of glucose metabolism activates the hexosamine pathway, whose end product is GlcNAc [42]. The long-term treatment of 5xFAD mice with the selective OGA inhibitor leads to significant reductions in Aβ accumulation and blocks cognitive impairments in AD model mice [43], as has also been reported elsewhere [44, 45].

With regard to *tau*, multiple studies have reported that OGA inhibition leads to reduced tauopathy and cerebrospinal fluid *tau* in AD models [46, 47]. The mechanism(s) underlying increased tau *O*-GlcNAcylation leading to less *tau* aggregation may involve the destabilization of nucleation filaments, increased *tau* solubility [48], as well as the blockade of pathological *tau* hyperphosphorylation [49]. The addition of *O*-GlcNAc to protein substrates is mediated by a glycosyltransferase called protein *O*-GlcNAc transferase (OGT), which belongs to GT41 (EC 2.4.1.255). OGT uses uridine 5’-diphospho-N-acetylglucosamine (UDP-GlcNAc) as a donor sugar to transfer GlcNAc to Ser or Thr residues [50]. Furthermore, α-acetylglucosaminidase (EC 3.2.1.50), which belongs to GH89, catalyzes the transferase cleavage of UDP off of redundant UDP-GlcNAc [51]. We found that the proportion of GH84 and GH89 in the SAMR1 group here was significantly lower than in the SAMP8 group. Furthermore, there was no significant difference in the proportion of GT41 in the SAMR1 and SAMP8 groups in the present study, indicating that protein (APP or *tau*) and *O*-GlcNAc levels in the SAMP8 group may be lower than in the SAMR1 group.

It is critical to emphasize that amyloid plaques contain multiple molecules beyond Aβ [52, 53]. For instance, chitin-like deposits have been found in sporadic AD by Calcofluor staining [54]. Chitin is a homopolymer of β1-4-linked GlcNAc and is synthesized by chitin synthase (EC 2.4.1.16) or hyaluronan synthase (EC 2.4.1.212) (both belong to GT2) [55]. Recent evidence suggests that polymerization of GlcNAc, together with Aβ and *tau*, may contribute to neuronal damage in sporadic cases of AD. GlcNAc polymers further exhibit significant neurotoxicity *in* *vitro* and lead to synaptic impairments and decreased levels of syntaxin and synaptophysin, reducing long-term potentiation at excitatory synapses [56]. Furthermore, GlcNAc cleaves the chitobiose core of N-linked glycans by a glycoside hydrolase called endoglycosidase H (EC 3.2.1.96) [57], which belongs to the GH85 family. Notably, GH85 was the only glycoside hydrolase family the proportion of which was lower in the SAMP8 group than in the SAMR1 group, a trend that was reversed with CA-30 administration. In the present study, we report, in detail, changes in CAZymes in SAM mice and those that occur with CA-30 treatment. CA-30 facilitated advantageous SAMR1-like changes in CAZymes of SAMP8 mice and may be principally relevant to the modulation of GH family-related changes, especially in GH85.

The results described here implicate intestinal microflora (including *Bacteroides*, *Prevotella*, *Parabacteroides*, *Clostridium*, *Ruminococcus*, and *Acidiphilium*) in controlling AD-like NIM network and cognition changes. In mammalian systems, the neural, endocrine, and immune systems influence one another to achieve homeostasis [58]. In the present study, we find that levels of GnRH and LH in the HPG axis are mostly positively correlated with alterations in microbiota composition. Epidemiological and biochemical studies indicate an association between hormones implicated in the HPG axis and cognitive senescence [59]. In particular, changes in HPG hormones are involved in the cognitive decline, mood disorders, and Aβ metabolism changes observed in AD [60–62]. All hormones and receptors in this axis have known functions in regulating neuron development and adult brain structure. Critically, receptors for these hormones are concentrated in the pyramidal neurons of the hippocampus, which is particularly vulnerable to AD pathology [63].

The immune system maintains homeostasis at the luminal surface of the intestinal host-microbial interface, which is critical for maintaining animal health [64]. Indeed, the immune system communicates bidirectionally with the CNS and is, therefore, a prime pathway for communicating the effects of gut microbiota to the CNS [65, 66]. Additionally, neuroinflammation is a key pathological hallmark of AD, with inflammatory cytokines actively contributing to neurodegeneration and Aβ plaque accumulation [67–69]. For instance, IL-4, an anti-inflammatory cytokine, is decreased in AD models and postmortem AD human brain tissues [70]. The beneficial effects of increased IL-4 in the hippocampus on reducing astro/microgliosis, Aβ oligomerization, enhancing neurogenesis, and improving spatial learning in an AD mouse model have also been reported [71].

Other cytokines have also been implicated in AD pathogenesis. For example, IL-5 derived from Th2 cells acts as an anti-inflammatory immune mediator and is significantly decreased in the peripheral blood of AD patients [72]. Additionally, the increased TNF-α signaling may lead to synaptic and neuronal hyperexcitability by modulating the expression of sodium and potassium channels [73, 74]. Inhibiting TNF-α signaling before amyloidosis further prevents the development of synaptic deficits in an AD mouse model [75]. IFN-γ, a pleiotropic pro-inflammatory cytokine, is unchanged in AD patients and enables the upregulation of glial and neural MHC class II proteins [76]. In addition, overexpression of IFN-γ results in robust suppression of APP production and metabolism [77], consistent with the observation that IFN-γ suppresses Aβ deposition via interactions with activated glia and promotes T cell-mediated immune infiltrates after Aβ immunization [78, 79].

The present study revealed that, at the genus level, *Bacteroidetes* (*Bacteroides, Prevotella, Parabacteroides, Odoribacter*), *Firmicutes* (*Clostridium, Ruminococcus, Butyrivibrio*), and *Proteobacteria* (*Acidiphilium*) were positively or negatively correlated with the immune system and cognitive performance changes. Similarly, *Bacteroides* [80], *Prevotella* [80, 81], and *Clostridium* [82] were closely correlated with cognitive abilities in prior studies. Moreover, the relative abundance of *Bacteroides* [83, 84], *Prevotella* [95], and *Ruminococcus* [85] were correlated with levels of inflammatory cytokines. Inversely, microbial infection and prebiotics may regulate levels of circulating cytokines, which have an obvious effect on brain function [86–88]. Furthermore, our finding that the relative abundances of *Bacteroides*, *Parabacteroides*, *Prevotella*, and *Ruminococcus* are balanced by CA-30 treatment in the present study is, to the best of our knowledge, entirely novel. No other reports on these genera have correlated them with cognitive performance or cytokine or chemokine levels, as in the present study.

Taken together, our comparison of the dominant genus (*Bacteroides*, *Prevotella*, *Parabacteroides*, *Odoribacter*, *Acidiphilium*, and *Ruminococcus*) is closely correlated with the abnormal secretion of cytokines. Abnormal cytokine secretion negatively regulates immune responses by suppressing Th cell responses [89], inhibiting Treg differentiation [90, 91], and inducing T cell anergy and apoptosis [92, 93]. Our finding that an increased number of Tregs are evident in SAMP8 mice is poorly understood and inconsistent with previous observations. Previous studies have suggested that Tregs increase with age and exert intensified suppressive activity in AD patients [94]. In contrast, an overall decreased frequency of Tregs has been described in mild AD patients as compared to age-matched controls [95]. Another study reported that the depletion of Tregs is accelerated by the onset of cognitive deficits triggered by amyloid deposition in a mouse model of AD [96]. Our finding that reduced numbers of B cells are present in SAMP8 mice is consistent with previous observations in AD patients [97, 98] and may be a consequence of T cell deficits in these mice and individuals. It is likely that the depletion of B cells and their activation by T cells leads to a loss of adaptive innate immune cross talk and thus accelerated AD progression [99].

In summarizing the findings of the present report, it is clear that CA-30 primarily works to alter the abundance of *Acidiphilium*, *Ruminococcus*, four genera in the SAMR1 and CA-30 groups (*Thermomonas*, *Paramesorhizobium*, *Alicycliphilus*, and *Candidatus_Midichloria*), and 10 newborn genera in the CA-30 group (*Xylophilus*, *Serpens*, *Hathewaya*, *Tanticharoenia*, etc.). These changes were evidenced by GH85 changes; rebalanced cytokines, hormones, and lymphocyte subsets in the NIM network; and slowed aging processes and decreased cognitive impairments in SAMP8 mice. These findings enhance our understanding of the pharmacological association between CA-30 and dysbiosis of the gut-brain axis and suggest that modulation of the intestinal microbiome is a potential intervention for protecting against the progression of AD. The present study highlights the possibility that CA-30 produces a synergistic effect on intestinal microbiome dysregulation and gut-brain axis malformations, though the precise mechanisms underlying the anti-AD effects of CA-30 require further investigation. Summarizing the present findings supports the use of CA-30 as a potential therapeutic agent for AD.

## MATERIALS AND METHODS

### Preparation of CA-30

CA-30 was prepared from the Liuwei Dihuang decoction (LW), a traditional Chinese medical preparation. LW was prepared as we have previously described [29, 30]. LW was decocted in 10 volumes of deionized water by two, two-hour boiling reflux treatments. After finishing this extraction, the materials were filtered through six-layer gauze to yield three extraction solutions which were then centrifuged. The supernatant was concentrated and then extracted in ethanol to produce LWD. The water elution fraction of the LWD was dissolved using an active carbon absorption column to obtain CA-30, the oligosaccharide component of LW. The CA-30 component was analyzed using high-performance liquid chromatography. Briefly, for the CA-30 component, the chromatographic separation was obtained on a diamond C18 column. This CA-30 component is mainly composed of stachyose and mannotriose [18].

### Experimental animals

Original SAMR1 and SAMP8 mice were generously provided by Dr. T. Takeda at Kyoto University, Japan. Mice were maintained at the Beijing Institute of Pharmacology and Toxicology under standard housing conditions (room temperature 22±1°C and humidity of 55±5%) on a 12-h light/dark cycle and were provided food pellets and water (provided by the Animal Center of the Beijing Institute of Pharmacology and Toxicology). They were allowed free access to water and food. Three or four mice were kept in each cage. Six-month-old male SAMP8 mice were randomly divided into two groups of 11 mice each.

**Figure 9:**
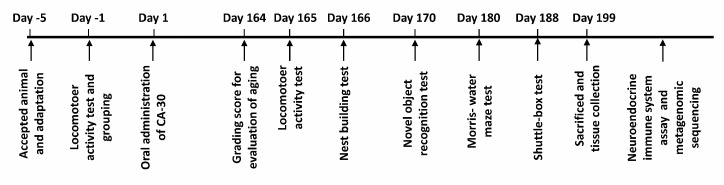
**The schematic diagram of the experimental procedure.**

CA-30 was dissolved in distilled water at 122.4 mg/mL. The drug-treated mice group was given an intragastric administration of CA-30 (0.1 mL/10 g body weight) once daily for 199 days. SAMP8 mice were age-matched to SAMR1 mice (9 males) as a control group and administered an equal volume of deionized water. Mice were weighed every three days. Drug administration and behavioral tests were performed according to predetermined experimental timelines (Figure 9). After the behavioral experiments, mice were placed in a sealed chamber and euthanized *via* isoflurane inhalation followed by cervical dislocation. The whole brain, hippocampus, cortex, hypothalamus, pituitary, plasma, and stool from each mouse were collected for hormone determination, lymphocytes subset analyses, and cytokine and metagenomic analyses.

### Evaluation of senescence

For evaluation of the degree of senescence in the SAMP8 mice, a grading score system developed by Hosokawa, M., et al. (1984) [100] was adopted. In brief, this scoring system, which was designed to assess changes in animal behavior and appearance, included measures of reactivity, passivity, glossiness, coarseness, hair loss, ulcer, periophthalmic lesions, cataract formation, corneal ulcers, corneal opacity, and lordoscoliosis. Grade 0 represented no notable changes and grade 4 represented the most severe changes.

### Behavioral assessments

#### Nest building

The procedure for the nest building test employed here was previously described by Deacon, et al. (2006) [101]. Briefly, mice were placed into individual testing cages with one nestled (5-cm squares of cotton batting) in each cage at day 166. Nests were assessed the next morning using a pre-determined rating scale, least being 0, best being 5.

#### Locomotor activity

Locomotor activity was assessed via the protocol outlined by Wang, et al. (2016) [18]. Motor behavior was tracked with a video-based behavior monitoring system (XinRuan Software Technology Co. Ltd., Shanghai, China). Each mouse was recorded for 20 min and its total distance traveled was calculated.

#### Novel object recognition

The object recognition test was administered as described by Rick Bevins and Toyce Besheer (2006) [102]. This procedure included three phases: habituation, training, and testing. To habituate them to the testing environment, animals freely explored a vacant chamber (20 min daily) for two consecutive days. On the third day, two of the same objects (sample objects A and B) were placed into the chamber. Each mouse was then allowed to explore the objects for 16 min. The training-to-testing intervals were 1 and 24 h. After a 1 h training-to-testing interval, mice were placed back into the chamber and one of the two identical objects was replaced with a novel object (novel object C). A 4 min testing session was completed to assess short-term object recognition memory. On the fourth day, after a 24 h training-to-testing interval, mice were again allowed to freely explore novel object D to test their long-term object recognition memory. A preferential index (time on novel object C/(time on novel object C+time on sample object A)×100%) was the computed to assess both short- and long-term object recognition memory.

#### Morris-water maze

The Morris water maze test protocol described by Charles V Vorhees & Michael T Williams (2006) [103] and Wang, et al. (2016) [18] was employed here. In brief, mice completed four daily trials in the presence of a hidden platform for six consecutive days. When the mouse located the platform, it was allowed to remain there for 5 s. If the mouse was unable to locate the platform within 60 s, it was placed onto the platform for 5 s to familiarize itself with the platform’s location. In probe trials, the platform was removed and the mouse was allowed to search for it for 60 s. The latency to reach the hidden platform in training and probe trial sessions, the number of crossing over the removed platform location, and the time spent in the target (platform) quadrant were recorded and analyzed.

#### Shuttle-box test

The shuttle-box test protocol described by Cheng, et al. (2011) [104] was utilized here. Briefly, working memory was evaluated using the shuttle-box apparatus (XinRuan Software Technology Co. Ltd., Shanghai, China). Each testing session began with acclimatization to the chambers for 2 min, followed by 30 trails with an inter-trial interval of 30 s. A tone (60 dB) and light (8 W) were co-administered as conditioned stimuli for 10 s each. An electrical foot shock (0.2 mA) was used as the unconditioned stimulus and was administered for 5 s following the presentation of the conditioned stimuli. This shuttle-box procedure was performed for a total of nine days. The software scored active avoidance behavior. On the 10^th^ day, all mice were submitted to another session in the shuttle-box in which the conditioned stimulus was not proceeded by a shock. The number of successful active avoidance times was recorded.

### Hormone radioimmunoassay

Hypothalamus and pituitaries were weighed and boiled separately in 1 mL of saline for 5 min. Peptides were extracted from hypothalamus and pituitary homogenates in 0.5 mL of 1 M glacial acetic acid, followed by centrifuging at 3000 rpm for 30 min. Concentrations of corticotropin releasing hormone (CRH), gonadotropin-releasing hormone (GnRH) and thyrotrophic hormone releasing hormone (TSH) in the supernatants of hypothalamus, adrenocorticotropic hormone (ACTH), luteinizing hormone (LH), follicle-stimulating hormone (FSH), thyroid stimulating hormone (TSH), and growth hormone (GH) in the supernatants of pituitary, corticosterone, testosterone (T), estradiol (E_2_), triiodothyronine (T3), and thyroxine (T4) in the plasma were determined with ^125^I-CRH RIA (Department of Neurobiology of the Second Military Medical University, Shanghai, China), ^125^I-GnRH RIA (Department of Neurobiology of the Second Military Medical University), ^125^I-TRH RIA (North Institute of Biological Technology), ^125^I-ATCH RIA (North Institute of Biological Technology, Beijing, China), ^125^I-LH RIA (North Institute of Biological Technology), ^125^I-FSH RIA (North Institute of Biological Technology), ^125^I-TSH RIA (North Institute of Biological Technology), ^125^I-GH RIA (North Institute of Biological Technology), ^125^I-CORT RIA (North Institute of Biological Technology), ^125^I-T RIA (North Institute of Biological Technology), ^125^I-E_2_ RIA (North Institute of Biological Technology), ^125^I-T3 RIA (North Institute of Biological Technology), and ^125^I-T4 RIA (North Institute of Biological Technology) kits, respectively.

### Flow cytometric analyses

Mouse blood cells were harvested and divided into three parts. First, spleen cells were treated with 100 μL of 20 μg/mL FITC anti-mouse CD3 antibody (100306, BioLegend, San Diego, USA), 100 μL of 12.5 μg/mL Percp anti-mouse CD4 antibody (100407, BioLegend), and 100 μL of 12.5 μg/mL APC anti-mouse CD8 antibody (100732, BioLegend) at 25°C for 30 min, washed, then incubated with 100 μL of 10 μg/mL FITC goat anti-mouse IgG antibody (405305, BioLegend) at 25°C for 30 min in the dark. Second, blood cells were treated with 100 μL of 12.5 μg/mL Percp anti-mouse CD4 antibody (BioLegend), 100 μL of 25 μg/mL APC anti-mouse CD25 antibody (101910, BioLegend), and 100 μL of 12.5 μg/mL PE anti-mouse Foxp3 antibody (320008, BioLegend) via the same protocol described above. Third, blood cells were treated with 100 μL of 20 μg/mL FITC anti-mouse CD19 antibody (115506, BioLegend) using the same protocol described above. After incubation, cells were washed and resuspended in 0.5 mL of PBS/2% paraformaldehyde and quantified by flow cytometry (BD Calibur^TM^).

### Multiplex bead analyses

Mouse plasma samples were diluted 1:2 in assay buffer and analyzed via a multiplex bead analysis approach. Interleukin-1β (IL-1β), IL-2, IL-5, IL-17, IL-6, IL-4, IL-10, granulocyte-macrophage colony stimulating factor (GM-CSF), granulocyte colony stimulating factor (G-CSF), interferon-γ (IFNγ), tumor necrosis factor α (TNF-α), monocyte chemotactic protein-1 (MCP-1), regulated upon activation normal T cell expressed and secreted factor (RANTES), Eotaxin, Macrophage Inflammatory Protein-1β (MIP-1β), IL-23, and TNF-β were measured according to the manufacturer’s instructions (Millipore Corp., USA). All samples were analyzed on the Luminex 200^TM^ (Luminex, Austin, TX, USA) platform.

### Genomic analyses

#### DNA extraction, library construction, and metagenomic sequencing

All fecal specimens were freely defecated by mice and then immediately collected. A total of 180-220 mg of fresh stool was collected from each mouse at day 198. Total genomic DNA was extracted using the E.Z.N.A.® DNA Kit (Omega Bio-Tek, Norcross, GA, U.S.), according to the manufacturer’s instructions. The concentration and purity of the extracted DNA were determined with a TBS-380 mini-fluorometer (Turner Biosystems, Sunnyvale, USA) and NanoDrop 2000 spectrophotometer (Thermo Scientific, Wilmington, USA), respectively. DNA extract quality was also checked on a 1% agarose gel. DNA extract was fragmented to an average size of about 300 base pairs using Covaris M220 (Gene Company Limited, China) for paired-end library construction. The paired-end library was constructed using a TruSeqTM DNA Sample Prep Kit (Illumina Inc., San Diego, USA). Adapters containing the full complement of sequencing primer hybridization sites were ligated to the blunt-ends of fragments. Paired-end sequencing was performed on the Illumina HiSeq4000 platform (Illumina Inc., San Diego, USA) at Majorbio Bio-Pharm Technology Co., Ltd. (Shanghai, China) and utilizing the HiSeq 3000/4000 PE Cluster and HiSeq 3000/4000 SBS kits, according to manufacturer instructions.

#### Sequence quality control and genome assembly

Adapter sequences were stripped from the 3' and 5' ends of paired-end Illumina reads using SeqPrep. Low-quality reads (length <50 bp or with a quality value <20 or having N bases) were removed by Sickle (https://github.com/najoshi/sickle). Metagenomics data were assembled using MEGAHIT [105], which makes use of succinct de Bruijn graphs. Contigs with a length over 300 bp were selected for final assembly and used for further gene prediction and annotation.

#### Gene prediction, taxonomy, and CAZy analyses

Open reading frames (ORFs) from each assembled contig were predicted using MetaGene (www.metagene.de) [106]. ORFs with a predicted length of over 100 bp were retrieved and translated into amino acid sequences using the NCBI translation table. All predicted genes with a 95% sequence identity (90% coverage) were clustered using cluster database at high identity with tolerance (CD-HIT, http://weizhongli-lab.org/cd-hit/) [107] with the longest sequences from each cluster selected as representative for the construction of a non-redundant gene catalog. Reads after quality control were mapped to representative sequences with 95% identity using short oligonucleotide analysis package (SOAP, https://anaconda.org/bioconda/soapaligner) [108] and gene abundance in each sample was evaluated. Representative sequences of non-redundant gene catalogs were aligned to the NCBI NR database with an e-value cutoff of 1e^-5^ using the basic local alignment search tool (BLAST, https://blast.ncbi.nlm.nih.gov/Blast.cgi) [109] for taxonomic annotations. Clusters of carbohydrate-active enzymes (CAZymes) were annotated for their representative sequences using BLAST against a carbohydrate-active enzyme database [32, 110] and with an e-value cutoff of 1e^-6^.

### Statistical analyses

All data were expressed as means ± S.D. GraphPad Prism 7.0 (GraphPad Software, Inc., La Jolla, CA, USA) was utilized to plot and analyze all data. Data from the two groups were compared by Student's *t*-tests. Inter-group differential taxonomic ranks (from phylum to genus) were detected using a student’s test in R (v3.1.2). Spearman correlation coefficients (R, v3.1.2) were used to measure correlations between differentially abundant genera and mouse behavioral outcomes. *P*<0.05 indicated statistical significance.

## Supplementary Material

Supplementary Figures
