# Supplementary material for: CA-30, an oligosaccharide fraction derived from Liuwei Dihuang decoction, ameliorates cognitive deterioration via the intestinal microbiome in the senescence-accelerated mouse prone 8 strain
Source: Aging (Albany NY). 2019 Jun 3;11(11):3463–86. doi: 10.18632/aging.101990 (PMC6594795; doi:10.18632/aging.101990)
Supplement: Supplementary Figures [file aging-11-101990-s001.pdf]

SUPPLEMENTARY FIGURES

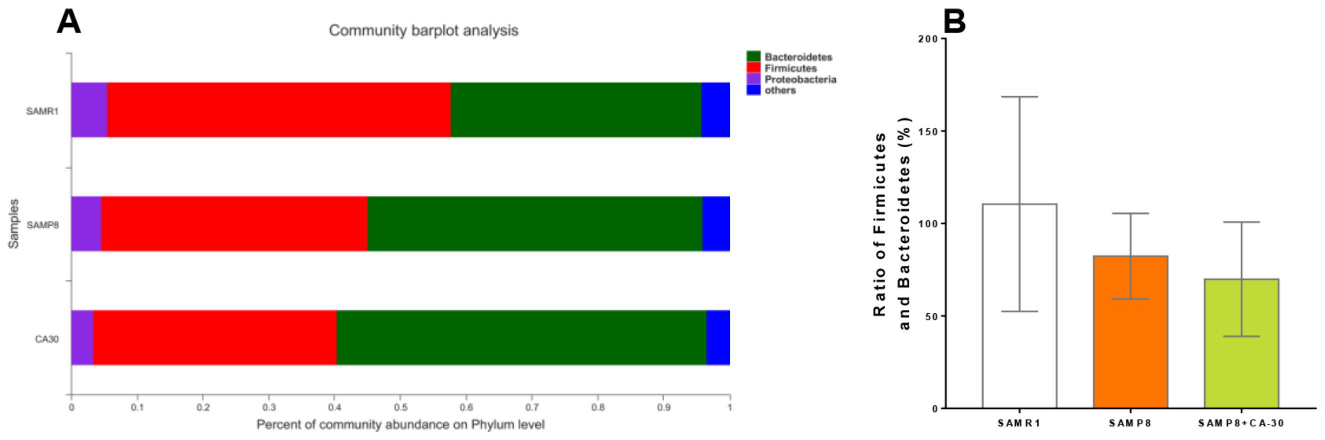

Supplementary Figure 1. Dominant bacteria taxa at the Phylum (**A**) level and abundant ratio of Firmicutes and Bacteroidetes (**B**) from all samples.

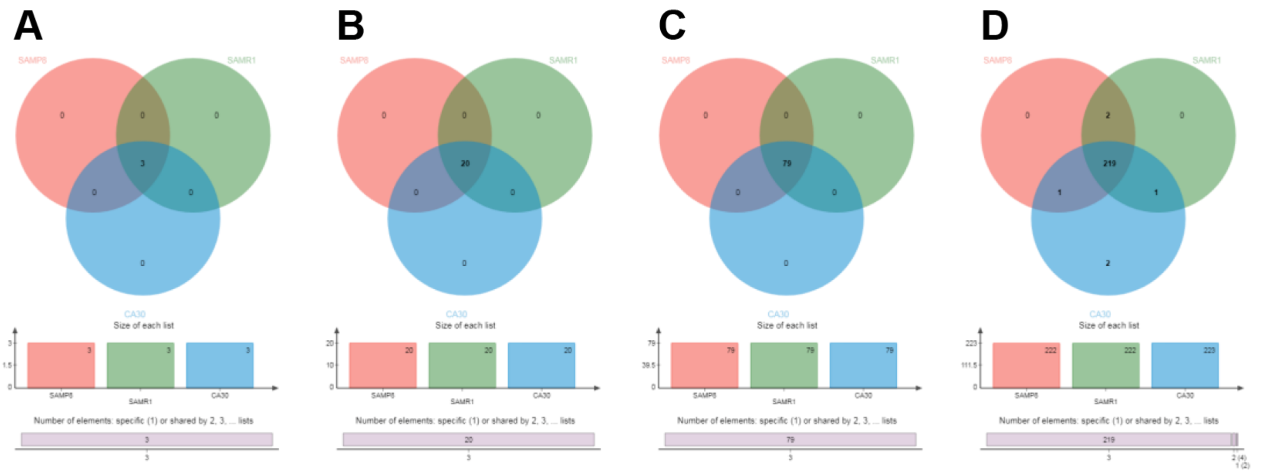

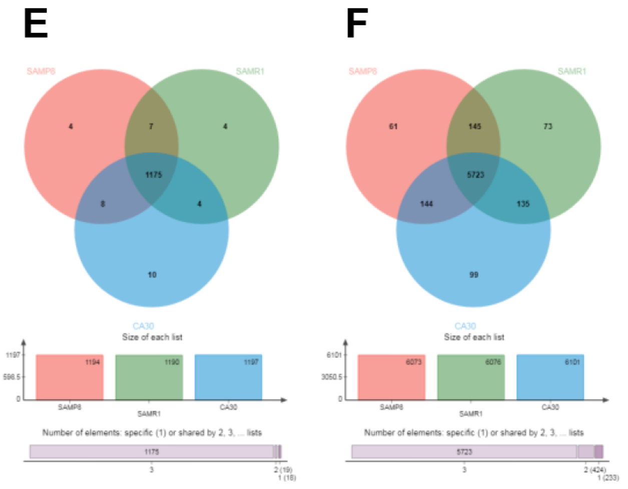

G

Table 1 Summary of gut microbiota composition in all mice

Group Label	Number					
	Phylum	Class	Order	Family	Genus	Species
SAMR1 & SAMP8 & CA30	3	20	79	219	1175	5723
SAMR1 & SAMP8	3	20	79	221	1182	5868
SAMR1 & CA30	3	20	79	220	1179	5858
SAMP8 & CA30	3	20	79	220	1183	5867
SAMR1 only	0	0	0	0	4	73
SAMP8 only	0	0	0	0	4	61
CA30 only	0	0	0	2	10	99

Supplementary Figure 2. Venn diagram of bacteria taxa at the Phylum (**A**), Class (**B**), Order (**C**), Family (**D**), Genus (**E**) and Species (**F**) level from all samples, and the summary of gut microbiota composition in all mice.

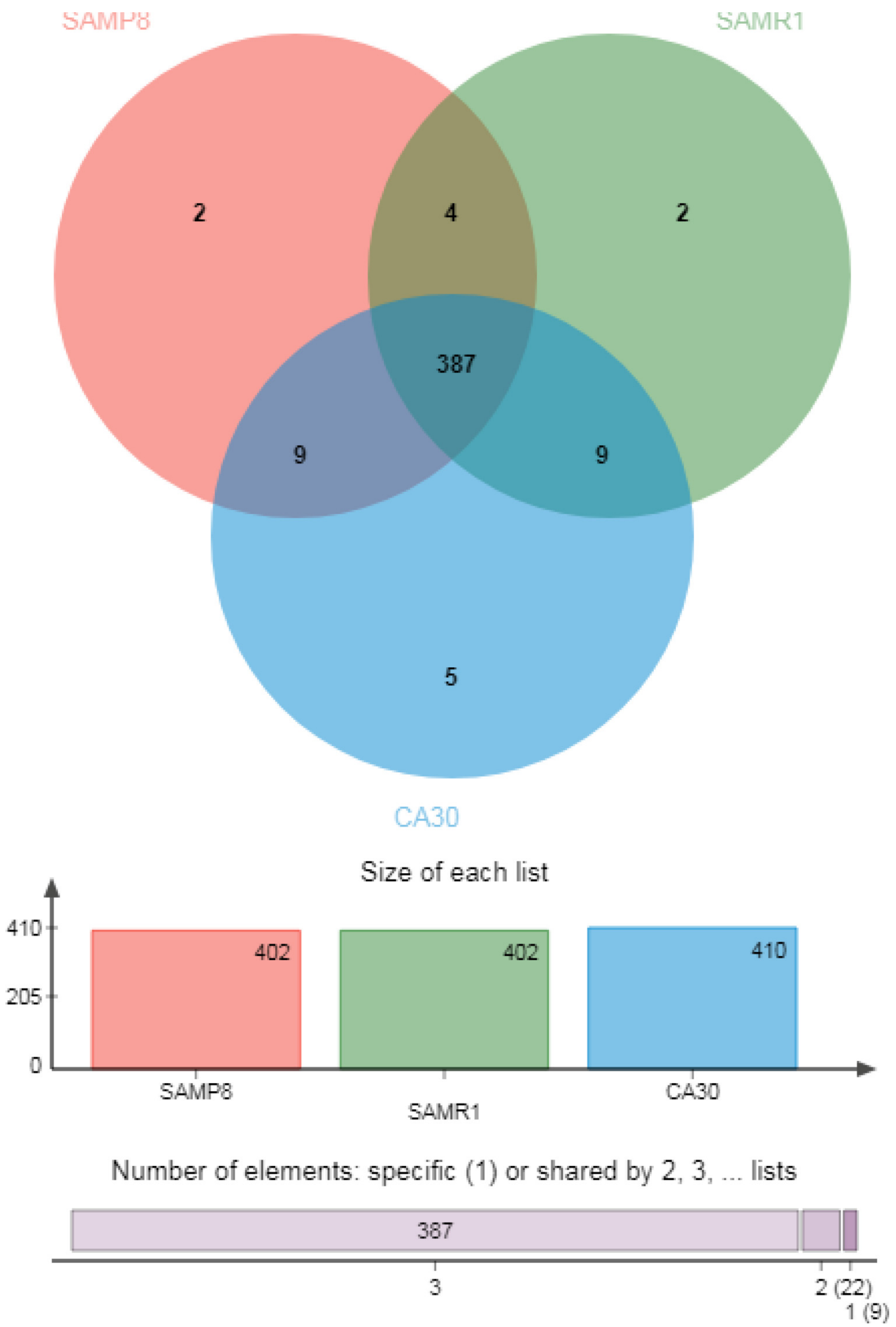

Supplementary Figure 3. Venn diagram of microbial carbohydrate-active enzymes in all mice. A total of 402, 402, and 410 non-redundant microbial carbohydrate-active enzymes were assigned for metaproteomes of SAMR1, SAMP8, and CA-30 group, respectively.
